# Integrating Traditional Wisdom With Modern Science: Ginsenosides as Antiheart Failure Agents

**DOI:** 10.1155/cdr/3078311

**Published:** 2026-04-26

**Authors:** Li Zhou, Yanhua Li, Wenjie Yin, Xi Gu, Jiana Shi, Fan Yang

**Affiliations:** ^1^ Department of Pharmacy, Yan′an Hospital Affiliated to Kunming Medical University, Kunming, Yunnan, China; ^2^ Center for Clinical Pharmacy, Cancer Center, Department of Pharmacy, Zhejiang Provincial People′s Hospital (Affiliated People′s Hospital, Hangzhou Medical College), Hangzhou, Zhejiang, China; ^3^ Joint Surgery, The General Hospital of Tibet Military Region, Lhasa, Tibet, China

**Keywords:** biomedical, Chinese traditional, ginsenosides, heart failure, medicine, oxidative stress, translational science, ventricular remodeling

## Abstract

Heart failure (HF), as the terminal stage of various cardiovascular diseases, remains a global health challenge with persistently high morbidity and mortality rates. Although current therapies—including angiotensin receptor–neprilysin inhibitors, sodium‐glucose cotransporter‐2 inhibitors, and cardiac resynchronization therapy—have improved outcomes, significant limitations persist. Ginseng, a renowned traditional Chinese medicine, contains bioactive ginsenosides (e.g., Rb1, Rb3, Re, Rg1, and Rg3) that exhibit multitarget cardioprotective effects, offering a promising therapeutic strategy for HF. This review systematically elucidates the mechanisms by which ginsenosides ameliorate HF, including modulation of myocardial energy metabolism, suppression of oxidative stress and inflammation, attenuation of fibrosis, and inhibition of cardiomyocyte apoptosis. We further evaluate the synergistic efficacy of ginsenoside‐containing formulations (e.g., YiQiFuMai injection and Qiliqiangxin capsule) in HF management. Although clinical evidence remains limited and heterogeneous, preclinical studies robustly support ginsenosides as pleiotropic natural compounds with therapeutic potential for HF. Future research should prioritize high‐quality clinical trials, optimize delivery systems (e.g., nanocarriers), and explore combination therapies with conventional drugs to accelerate clinical translation.

## 1. Introduction

Heart failure (HF) represents a complex clinical syndrome characterized by structural and/or functional cardiac abnormalities, leading to impaired ventricular systolic and/or diastolic function. As the terminal stage of various cardiovascular diseases, HF is marked by diminished cardiac output and inadequate peripheral tissue perfusion [1]. With the aging population and increasing prevalence of cardiovascular diseases, HF has emerged as a major global public health challenge, exhibiting escalating incidence, readmission rates, and mortality [2]. Consequently, therapeutic strategies focusing on mortality reduction, readmission prevention, and quality‐of‐life improvement have become paramount in both clinical practice and scientific research, driving the translation of basic research into clinical innovations. Although contemporary therapies, including pharmacological interventions like angiotensin receptor‐neprilysin inhibitors (ARNI) and sodium‐glucose cotransporter‐2 inhibitors (SGLT2i), as well as device‐based treatments such as cardiac resynchronization therapy and left ventricular assist devices, have significantly improved patient outcomes, limitations persist. These include drug‐related adverse effects, device dependency, and suboptimal responses in refractory HF cases [3, 4]. There remains an urgent need for novel therapeutic approaches, particularly multitarget agents addressing the complex pathophysiology of HF.

*Panax ginseng* C.A. Meyer (Araliaceae), a cornerstone of traditional Chinese medicine (TCM), has been used for millennia to treat “Qi deficiency” syndromes—a TCM pattern encompassing fatigue and palpitations, aligning with HF symptoms [5, 6]. To facilitate interdisciplinary understanding, it is important to clarify that “Qi” in TCM is not a substance per se, but a foundational concept representing the body′s vital energy and functional capacity. It governs physiological activities, including circulation, defense, and metabolism. Thus, “Qi deficiency” conceptually overlaps with the modern biomedical understanding of diminished systemic resilience and energy dysmetabolism, which are central to HF pathophysiology. This theoretical alignment provides a bridge between traditional use and contemporary scientific inquiry. Historical texts like Shennong Bencao Jing describe ginseng′s ability to “tonify Qi” and stabilize cardiac function [7], providing a direct link to its modern investigation for HF. Furthermore, modern pharmacological studies have identified ginsenosides as its primary bioactive constituents, responsible for diverse therapeutic effects including immunomodulation, metabolic regulation, antioxidative, antitumor, and cardioprotective activities [8, 9]. Emerging evidence suggests that ginsenosides and their formulations may ameliorate HF through multifaceted mechanisms: improving myocardial energetics, suppressing apoptosis, attenuating oxidative stress, and mitigating inflammatory responses [10]. Although prior reviews have documented ginsenosides′ cardioprotection in ischemia‐reperfusion models and COVID‐19–related cardiac complications [5, 11, 12], a systematic evaluation of their therapeutic potential specifically in HF, particularly their multitarget intervention capacity, remains lacking. This study therefore is aimed at critically analyzing current evidence on ginsenosides′ anti‐HF effects, bridging traditional knowledge with contemporary pharmacological insights. We hypothesize that these versatile compounds may offer novel therapeutic avenues for HF management.

A systematic search was conducted using PubMed, MEDLINE, and Web of Science with the core terms: (“Heart Failure” OR “Cardiac Dysfunction” OR “Myocardial Remodeling” OR “Low Cardiac Output Syndrome”) AND (“*Panax*” OR “Ginseng” OR “Ginsenosides”). From 107 initial records (2010–present) identified by title/abstract screening, 72 relevant publications were retained after deduplication and exclusion. This review synthesizes these findings to provide a foundation for future research and clinical development of ginsenoside‐based therapies.

## 2. Anti‐HF Effects of Ginsenosides

### 2.1. Classification of Ginsenosides

**Figure 1:**
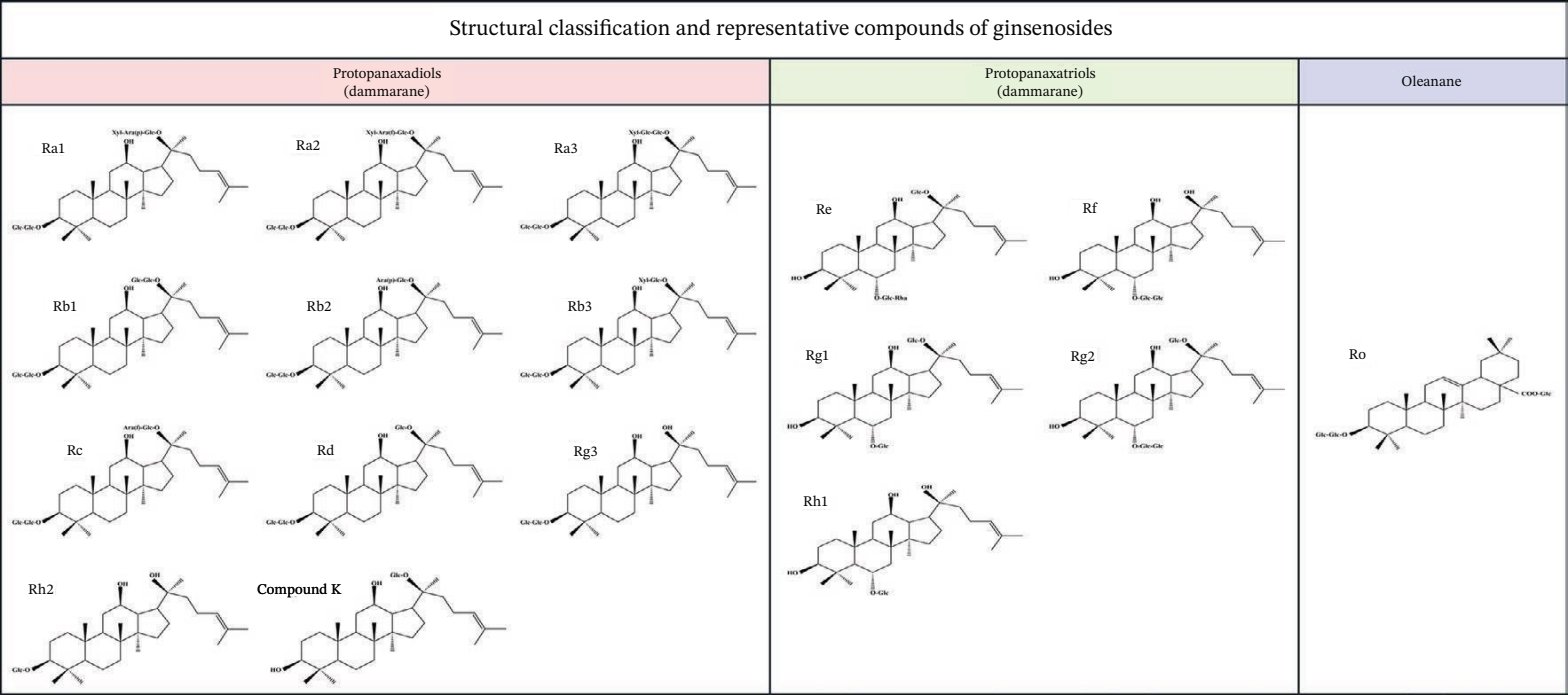
Structural classification of ginsenosides: protopanaxadiols, protopanaxatriols, and oleanane‐type.

Consistent with our previous research, ginsenosides can be structurally classified into three major types: protopanaxadiols (e.g., Ra1, Ra2, Rb1, Rb2, Rc, Rd, Rg3, Rh2, and compound K), protopanaxatriols (e.g., Re, Rf, Rg1, Rg2, and Rh1), and oleanane‐type (e.g., ginsenoside Ro). As illustrated in Figure 1, both protopanaxadiols and protopanaxatriols are derived from a dammarane‐type skeleton, differing in their glycosylation patterns—protopanaxadiols feature sugar moieties at the C‐3 position, whereas protopanaxatriols are glycosylated at C‐6. Notably, these two subtypes exhibit the most potent biological activities among ginsenosides, underpinning their pharmacological significance [13].

### 2.2. Therapeutic Effects of Major Ginsenosides on HF

In recent years, monomeric ginsenosides, particularly Rb1, Rb3, Re, Rg1, Rg2, and Rg3, have garnered significant attention for their therapeutic potential in HF. These bioactive compounds exert cardioprotective effects through multitarget mechanisms including suppression of myocardial remodeling and fibrosis, attenuation of oxidative stress and mitochondrial protection, anti‐inflammatory actions, improvement of energy metabolism disorders, promotion of angiogenesis and microcirculation, as well as modulation of cellular autophagy and apoptosis (Table 1).

**Table 1:** Therapeutic effects and main mechanisms of ginsenosides in heart failure.

Ginsenoside	Effects on HF	Mechanisms of Action	Experimental Models	Dosage/concentration	Potential applications/notes	References
**Rb1**	—Inhibits cardiomyocyte apoptosis; —attenuates cardiac remodeling; —improves energy metabolism disorders	—Suppresses PKA/caspase‐9 pathway; —modulates TGF‐*β*1/Smad and ERK/Akt signaling; —activates AMPK/PPAR*α* to enhance metabolic homeostasis	—ISO‐induced rat model; —pressure‐overload HF rat model; —H9c2 cell model	In vivo: 10–70 mg/kg/day (i.p., rodent models); 10 mg/kg (i.v., weekly, nanoparticle). In vitro: 0.1–100 *μ*M (cardiomyocytes); 1.25–100 *μ*g/mL (nanoparticles in cells).	—GRb1@PLGA nanoparticles improve bioavailability; —injectable hydrogel formulations under development	[14], [15], [16], [17]
**Rb3**	—Regulates myocardial energy metabolism; —suppresses cardiac fibrosis	—Activates PPAR*α* pathway; —modulates PI3K‐Akt/AMPK signaling	—In vitro/vivo HF models; —nanoparticle conjugation (NpRb3)	In vivo: 40 mg/kg/day (p.o.). In vitro: 20–80 *μ*M (cardiac fibroblasts)	Nanodelivery systems to enhance oral bioavailability.	[18–20]
**Re**	—Antifibrotic effects; —Reduces inflammation and ischemia‐reperfusion injury	—Activates eNOS/NO release; —inhibits TGF‐*β*1/Smad3 pathway; —regulates miR‐489/myd88/NF‐*κ*B axis	—ISO‐induced rat model; —acute MI mouse model	In vivo: 5–20 mg/kg/day (p.o.). In vitro: 50–200 *μ*M (cardiac fibroblasts)	Potential for coronary artery disease‐related HF.	[21–23]
**Rg1**	—Ameliorates mitochondrial dysfunction; —Antifibrotic and antiapoptotic effects	—Activates SIRT1/PINK1/Parkin–mediated mitophagy; —inhibits ERK1/2 phosphorylation; —modulates P2X7R‐Akt/GSK‐3*β* pathway	—LAD‐ligated mouse model; —sepsis‐induced cardiac dysfunction model	In vivo: 35–70 mg/kg/day (i.p.). In vitro: 25–100 *μ*M (H9c2 Cells)	Potential for age‐related HF.	[24], [25], [26]
**Rg2**	—Reduces myocardial fibrosis; —Improves post‐MI cardiac function	—Activates AKT phosphorylation in cardiac fibroblasts	—MI rat model; —in vitro cardiac fibroblast experiments	In vivo: 0.5 g/kg/day (i.g.). In vitro: 10 *μ*M (neonatal rat cardiac fibroblasts)	Candidate for preventing ventricular remodeling.	[27], [28]
**Rg3**	—Stabilizes Ca^2+^ homeostasis; —Antifibrotic effects; —Modulates energy metabolism	—Enhances SERCA2a‐SUMOylation; —inhibits TGFBR1 signaling; —activates AMPK/ULK1/FUNDC1 pathway	—TAC‐induced HF mouse model; —ISO‐induced HL‐1 cell hypertrophy model	In vivo: 20–40 mg/kg/day (i.g.). In vitro: 20 *μ*M (mouse cardiac fibroblasts)	—Rg3 micelles reduce doxorubicin cardiotoxicity; —patented formulations in development	[29], [30], [31], [32]
**Rb2**	—Reduces cardiomyocyte apoptosis and oxidative stress	—downregulates miR‐216a‐5p to enhance autophagy	—LAD‐ligated HF rat model; ‐ H9c2 cells under OGD/R	In vivo: 10–20 mg/kg/day (i.g.). In vitro:10–50 *μ*M (H9c2 cells)	Potential therapeutic target for HF	[33]
**Rd**	—Improves mitochondrial function; —Suppresses cardiac pathology	—Activates TBK1‐AMPK pathway; —modulates WNT5A/Ca^2+^ signaling	—TAC/CAL/ISO‐induced HF mouse models	In vivo: 5–20 mg/kg/day (i.g.). In vitro:5–20 *μ*M (adipocytes)	Cardioprotection via omentin secretion from adipocytes	[34]
**Rg5/Rh2**	—Suppresses cardiac inflammation and remodeling; —Attenuates Ang II‐induced hypertrophy	—Blocks JNK/AP‐1 pathway; —inhibits TLR4‐NF‐*κ*B inflammatory signaling	—Ang II–induced hypertensive HF mouse model	In vivo: 20–50 mg/kg/day (i.g.). In vitro:5–10 *μ*M (neonatal rat ventricular myocytes)	Suitable for HF with comorbid hypertension	[35], [36]
**Synergistic Effects**	—Multitarget HF improvement (anti ‐ inflammatory + metabolic + antifibrotic)	—Coordinated modulation of PI3K‐Akt, AMPK, and TGF‐*β* pathways	—Zebrafish HF model; —Network pharmacology analysis	Pharmacodynamic markers: Rg3: 25–100 *μ*g/mL; Rg5: 0.25–1 *μ*g/mL; Rg6: 25–100 *μ*g/mL	Development direction for ginseng polypharmaceuticals (e.g., Rg1 + Rg3 + Rb1)	[37–39]

Abbreviations: i.p., intraperitoneal; i.g., intragastric; i.v., intravenous; p.o., per os (oral gavage).

### 2.3. Anti‐HF Effects of Ginsenoside Rb1

Cardiomyocyte apoptosis and pathological autophagy represent critical pathways in HF pathogenesis. Ginsenoside Rb1, a major active component isolated from *P*. *ginseng* and *Panax notoginseng*, has been demonstrated to reduce isoproterenol (ISO)–induced cardiomyocyte apoptosis both in vitro and in vivo, potentially through inhibition of protein kinase A and caspase‐9 pathways [14]. In pressure‐overload HF rat models, Rb1 was shown to suppress excessive autophagy via modulation of Rho/ROCK and PI3K/mTOR signaling cascades [40].

The “antiremodeling/prosurvival” dual effects constitute a multitarget strategy for HF treatment. Comparative studies using losartan (4.5 mg/kg) as positive control revealed that Rb1 administration (35–70 mg/kg for 8 weeks post‐HF induction) improved cardiac function through downregulation of TGF‐*β*1/Smad and ERK pathways, activation of Akt signaling to restore mitochondrial function, and enhancement of glucose uptake to prevent adverse remodeling [15]. Furthermore, in doxorubicin‐induced chronic HF models, Rb1 ameliorated metabolic remodeling by improving myocardial fatty acid *β*‐oxidation via AMPK activation [41]. Another study confirmed that age‐related cardiac dysfunction was similarly attenuated by Rb1 through NF‐*κ*B–mediated suppression of fibrosis and inflammation [42].

Notably, the triad of anti‐inflammatory action, oxidative stress reduction, and mitochondrial function preservation serves as crucial therapeutic targets for HF management and progression delay. The anti‐inflammatory and mitochondrial protective properties of Rb1 were evidenced by its ability to counteract angiotensin II (Ang II)–induced cardiac hypertrophy and systemic inflammation, maintain mitochondrial function in cardiomyocytes under stress, and regulate the DUSP‐1‐TMBIM‐6‐VDAC1 axis to improve cardiac function [10, 43]. In addition, to enhance bioavailability, advanced Rb1 formulations have been developed. For example, ginsenoside Rb1‐PLGA nanoparticles alleviated oxidative stress–induced myocardial damage via ROS/PPAR*α*/PGC1*α* modulation in left anterior descending (LAD) ligated rats [16]; injectable Rb1/polydopamine hydrogels improved myocardial infarction (MI) outcomes through mitochondrial DNA‐STING crosstalk regulation [44].

Furthermore, the modulation of energy metabolism homeostasis and complementary signaling pathways has emerged as a critical therapeutic strategy in HF management. Accumulating evidence indicates that ginsenoside Rb1 exerts multifaceted metabolic regulatory effects, including [1] restoration of energy homeostasis through PPAR*α*‐mediated enhancement of ATP synthesis [17]; [2] inhibition of store‐operated Ca^2+^ entry, thereby attenuating pathological pulmonary vasoconstriction; and mitigation of right ventricular failure risk in pulmonary hypertension through integrated cardiometabolic protection [45].

### 2.4. Anti‐HF Effects of Ginsenoside Rb3

Emerging evidence has demonstrated the cardioprotective potential of ginsenoside Rb3 in HF. Experimental studies utilizing both in vitro and in vivo HF models have revealed that Rb3 ameliorates cardiac dysfunction primarily through PPAR*α* pathway activation, which modulates myocardial energy metabolism and attenuates cardiomyocyte apoptosis [18]. Notably, pharmacological optimization of Rb3 has been achieved through nanoparticle conjugation (NpRb3), significantly enhancing its oral bioavailability. This advanced formulation has shown superior efficacy in suppressing myocardial fibrosis via PPAR*α*‐mediated mechanisms, thereby offering a promising therapeutic strategy for HF management [19]. Recent systematic investigations employing network pharmacology and systems pharmacology approaches have further elucidated the mechanistic basis of Rb3′s anti‐HF effects. These studies identify Rb3, along with Rg1, as a lead therapeutic candidate that primarily targets the PI3K‐Akt and AMPK signaling pathways [20].

Collectively, the anti‐HF properties of ginsenoside Rb3 are mediated through three principal mechanisms: restoration of energy metabolic homeostasis, inhibition of cardiomyocyte apoptosis, and suppression of pathological myocardial fibrosis. This multitarget action profile positions Rb3 as a promising candidate for the development of novel HF therapeutics.

### 2.5. Anti‐HF Effects of Ginsenoside Re

Ginsenoside Re has demonstrated significant cardioprotective potential through multiple mechanisms. Early mechanistic studies revealed that Re acts as a specific agonist for nongenomic pathways of sex steroid receptors. Notably, it enhances endothelial NO synthase (eNOS) activity, leading to increased nitric oxide (NO) production, which serves as the molecular basis for cardiac K+ channel activation and protection against ischemia‐reperfusion injury [21]. In ISO‐induced myocardial fibrosis and HF models (5 mg/kg/day for 7 days), Re administration (5–20 mg/kg) significantly attenuated cardiac weight increase, myocardial fibrosis progression, and hydroxyproline content elevation. These effects were mediated through TGF‐*β*1/Smad3 signaling pathway modulation [22]. Additional studies using acute MI models and Ang II stimulated cardiac fibroblasts (CFs) demonstrated Re′s potent antifibrotic activity via regulating the miR‐489/myd88/NF‐*κ*B pathway [23]. Therefore, the synergistic anti‐inflammatory and antifibrotic effects position ginsenoside Re as a promising multitarget therapeutic agent for HF management, particularly for cases involving ischemia‐reperfusion injury, pressure‐overload induced remodeling, and metabolic cardiomyopathy.

### 2.6. Anti‐HF Effects of Ginsenoside Rg1 and Rg2

As protopanaxatriol‐type ginsenosides, both Rg1 and Rg2 have demonstrated significant therapeutic potential in HF through multifaceted mechanisms. Recent preclinical investigations using a rat chronic heart failure (CHF) model have systematically characterized the anti‐CHF effects of a compound mixture containing Rg1, Rg2, and notoginsenoside R1 through integrated pharmacodynamic, pharmacokinetic, and chemoprofile analyses. The therapeutic benefits were mediated by enhanced cardiac function, reduced N‐terminal pro‐B‐type natriuretic peptide (NT‐proBNP) levels, attenuated myocardial injury, and extracellular collagen degradation. Mechanistically, this combination promoted mitochondrial homeostasis primarily through heme synthesis inhibition and subsequent succinyl‐CoA accumulation [27]. Although research on Rg2 alone remains limited, a pivotal study demonstrated its ability to reduce post‐MI fibrosis in vivo, improve cardiac function, and also activate AKT phosphorylation in CFs. These findings position Rg2 as a promising antiremodeling agent for post‐MI ventricular reconstruction [28].

In contrast, Rg1 has been more extensively studied, with documented efficacy in multiple HF models. First of all, in pressure‐overload HF induced by transverse aortic constriction (TAC), Rg1 treatment significantly attenuated left ventricular hypertrophy. Mechanistic studies revealed this cardioprotective effect was mediated through coordinated activation of phosphorylated Akt and suppression of p38 mitogen‐activated protein kinase (MAPK) signaling pathways [46]. Secondly, in sepsis‐induced cardiomyopathy, Rg1 has been demonstrated to preserve mitochondrial function and mitigate cardiac dysfunction via the P2X7R‐Akt/GSK‐3*β* signaling pathway [24]. Moreover, in ischemic HF, Rg1 significantly improved cardiac remodeling in mice subjected to LAD artery ligation. This effect was attributed to enhanced SIRT1/PINK1/Parkin–mediated mitochondrial autophagy, which resulted in reduced left ventricular dilation and decreased cardiac fibrosis [25, 47]. Additionally, in doxorubicin‐induced CHF, Rg1 alleviated cardiac dysfunction by inhibiting the phosphorylation of ERK1/2, reducing apoptosis, promoting cell viability and proliferation, and attenuating cardiac inflammation [26]. Collectively, these findings position Rg1 as a multifunctional cardioprotective agent with particular promise for the management of HF with preserved ejection fraction, chemotherapy‐induced cardiotoxicity, and postischemic ventricular remodeling.

### 2.7. Anti‐HF Effects of Ginsenoside Rg3

Ginsenoside Rg3, one of the main active components of ginseng, possesses potent free radical scavenging ability and can exert cardioprotective effects. In recent years, reviews have summarized the pharmacological effects of Rg3 in the treatment of cardiovascular diseases and mental health disorders, and further explored its therapeutic potential for related diseases [48]. In HF mouse models and ISO‐induced HL‐1 cell hypertrophy models, original studies have found that Rg3 can effectively improve the abnormal Ca^2+^ homeostasis in HF, thereby protecting cardiac function, a process associated with the SUMOylation of sarcoplasmic/endoplasmic reticulum Ca^2+^ ATPase 2a [29]. Another study, through in vitro and in vivo experiments, found that Rg3 can improve myocardial glucose metabolism and insulin resistance by activating the AMPK signaling pathway, further confirming its cardioprotective effects on TAC induced HF [49]. Lai et al. further discovered through in vitro and in vivo HF experimental models that N‐acetylglutamine may be a potential biomarker for HF, and its specific metabolic degrading enzyme aminoacylase‐1 (ACY1) may be a potential therapeutic target for preventing and treating myocardial fibrosis during HF development. Moreover, Rg3 can reduce myocardial fibrosis and improve HF by increasing ACY1 expression and inhibiting the TGF‐*β*1/Smad3 pathway [30]. HF is usually not accompanied by increased glucose oxidation, which may be related to unchanged or reduced pyruvate oxidation in mitochondria. Therefore, some studies suggest that increasing pyruvate oxidation may be a new therapy for treating cardiovascular diseases, including HF. In TAC‐induced mouse myocardial hypertrophy models, it was found that Rg3 regulates myocardial pyruvate metabolism and reverses myocardial hypertrophy by inhibiting the acetyltransferase activity of P300 to reduce the level of dihydrolipoamide dehydrogenase 2‐hydroxyisobutyrylation [50]. In addition, based on the theory that enhancing mitochondrial autophagy can prevent pathological cardiac remodeling and HF, some studies have explored whether Rg3 can exert anti‐HF effects by activating mitochondrial autophagy through in vitro and in vivo experiments. The results showed that Rg3 activates mitochondrial autophagy by activating Unc51‐like Kinase 1 (ULK1) and inducing ULK1‐mediated FUNDC1 phosphorylation, thereby effectively treating HF [31]. Other studies have continuously focused on the issue of myocardial fibrosis–induced myocardial remodeling and HF after MI. They used LAD coronary artery ligation‐induced MI mouse models and TGF‐*β*1–stimulated primary CF models to confirm through in vivo and in vitro experiments that Rg3 exerts antimyocardial fibrosis and myocardial remodeling effects after MI by inactivating the TGFBR1 pathway and inhibiting CFs proliferation and collagen deposition, thereby alleviating HF [32]. Recently, a study integrated genomic information from multiple databases, constructed a protein–protein interaction network using drug–disease interaction genes, and combined ISO‐treated H9c2 cell experiments to verify the protective effects of Rg3 on HF, which may be achieved by inhibiting key genes in inflammatory signaling, including STAT3, CASP3, TNF, and IL‐6 [51]. Given the poor water solubility and low oral bioavailability of Rg3, researchers have encapsulated Rg3 through self‐assembly of Pluronic F127 to improve its solubility and oral bioavailability. Studies have confirmed that Rg3 micelles can reduce doxorubicin‐induced cardiotoxicity, improve mitochondrial and metabolic functions, and reduce reactive oxygen species production, thereby reducing the risk of doxorubicin‐induced HF. Meanwhile, Rg3 micelles have also enhanced the anticancer efficacy of doxorubicin [52].

In summary, the anti‐HF effects of ginsenoside Rg3 are mainly exerted through multiple pathways, including improving Ca^2+^ homeostasis, improving energy metabolism disorders, antimyocardial fibrosis, antioxidant stress and mitochondrial protection, and anti‐inflammation. Its multitarget action provides an effective theoretical basis and preclinical research evidence for the further development of drugs that can comprehensively resist HF.

## 3. Cardioprotective Effects of Other Ginsenosides in HF

Beyond the well‐characterized ginsenosides discussed above, emerging evidence suggests that several less‐studied ginsenosides may also possess significant therapeutic potential for HF. Although research on these compounds remains limited, preliminary findings warrant further investigation.

Ginsenoside Rb2 has demonstrated cardiovascular benefits, with one study revealing its ability to enhance autophagy while reducing apoptosis and oxidative stress in both LAD‐ligated HF rats and oxygen–glucose deprivation/reoxygenation (OGD/R)–treated H9c2 cells through miR‐216a‐5p downregulation [33]. Ginsenoside Rd has shown efficacy in improving cardiac function across multiple HF models, including TAC, coronary artery ligation, and ISO‐induced HF, via TBK1‐AMPK–mediated omentin secretion and WNT5A/Ca^2+^–dependent mitochondrial biogenesis [34]. Notably, ginsenoside Rg5 exhibits protective effects against Ang II–induced cardiac inflammation and remodeling through JNK/AP‐1 pathway inhibition, making it a promising candidate for hypertensive HF [35]. Similarly, ginsenoside Rh2 attenuates myocardial fibrosis, hypertrophy, and inflammation in Ang II–challenged mice without affecting blood pressure, primarily by suppressing JNK/AP‐1–associated inflammatory signaling [36]. Interestingly, innovative ginsenoside derivatives have also been developed, such as the highly bioavailable precursor 20S‐O‐GLC‐DM (C20DM), which outperforms metoprolol in ameliorating left ventricular diastolic dysfunction through enhanced mitochondrial quality control (PINK1/Parkin regulation), AMPK‐mTOR‐ULK1–mediated autophagy modulation, gut microbiota improvement, and TLR4‐MyD88‐NF‐*κ*B pathway inhibition [37, 38].

Furthermore, various ginsenosides have been identified as pharmacodynamic markers for different ginseng species. For instance, saponins including ginsenoside Rg1, Rg3, Rh2 and their derivatives were confirmed to mitigate anthracycline cardiotoxicity without compromising antitumor efficacy [39]. Moreover, several components including Rg3, Rg5, and Rg6 were found to serve as quality markers (Q‐markers) for American ginseng′s anti‐HF effects [53]. In another study, Rh4, Rk3, Rk1, Rg5 and compound K of ginseng targeted PI3K‐Akt, TNF, apoptosis, and mTOR pathways to exert anti‐HF effects [54]. In recent years, researchers screened out seven active components from red ginseng, including ginsenoside Rg1, Rg2, Rg3, Rb1, Rd, Re, and Ro, and demonstrated their cardioprotective effects in the OGD model [55]. In addition, disease module analyses further revealed that key ginsenosides, particularly Rh4 and Rg5, exert therapeutic effects in chronic HF through multitarget interactions with AKT1, TNF, and NFKB1, primarily via PI3K‐Akt and calcium signaling pathways [56].

Collectively, current research confirms that ginsenosides provide cardioprotection through both isolated compound actions and, more importantly, via synergistic network pharmacology effects. These findings position ginseng as a promising natural product for HF treatment, leveraging its unique “multicomponent–multitarget–multipathway” therapeutic paradigm that simultaneously modulates various pathological processes in HF progression.

## 4. Anti‐HF Effects of Ginsenosides in TCM Formulations

### 4.1. YiQiFuMai (YQFM) Injection

YQFM injection is a modern TCM formulation derived from the classic Shengmai San, containing red ginseng (*P*. *ginseng* C.A. Mey.), dwarf lilyturf tuber (*Ophiopogon japonicus* [L.f.] Ker Gawl.), and Chinese magnolia vine fruit (*Schisandra chinensis* [Turcz.] Baill.). It is primarily composed of ginsenosides Re, Rf, Rg2, and other bioactive compounds. It has been widely utilized as a classic and traditional therapeutic modality in clinical practice in China for the treatment of HF and angina pectoris. In LAD‐ligated CHF rat models, YQFM demonstrated comparable efficacy to captopril. In vitro studies have further identified that its ginsenoside components, including Rb1, Rg1, Rf, Rh1, Rc, Rb2, Ro, and Rg3, at effective concentrations around 100 *μ*M, exhibit potent anti‐inflammatory effects through NF‐*κ*B inhibition (particularly ginsenoside Ro) [57]. Mechanistic studies revealed YQFM′s cardioprotection involves MAPK pathway modulation, dual regulation of AMPK/PI3K‐Akt activation and MAPK suppression, omentin‐dependent protection mediated by ginsenoside Rd, adipocyte–cardiomyocyte crosstalk regulation [58, 59]. Moreover, a relevant study employing ultrafast liquid chromatography tandem mass spectrometry (UFLC‐MS/MS) pharmacokinetic analysis confirmed that these ginsenosides are the major bioactive components of YQFM [60].

### 4.2. Qiliqiangxin (QLQX) Capsule

The TCM QLQX capsule, a formulation comprising milkvetch root (*Astragalus membranaceus* [Fisch.] Bunge), ginseng (*P*. *ginseng* C.A. Mey.), prepared monkshood (*Aconitum carmichaelii* Debeaux), Danshen root (*Salvia miltiorrhiza* Bunge), and other herbs, is widely used in the clinical treatment of CHF. A growing body of research has confirmed that various ginsenosides, as the primary active components of QLQX, play a crucial role in its anti‐HF effects. One study on QLQX identified four major components, including ginsenoside Rg1, as suitable Q‐markers for QLQX in CHF treatment, which were directly associated with the inhibition of the activated renin–angiotensin–aldosterone system [61]. Another study proposed a novel multidimensional “radar chart” strategy to visually evaluate the Q‐markers of QLQX for its anti‐HF efficacy. The study revealed that seven compounds, including ginsenoside Re, served as high‐content Q‐markers of QLQX, exhibiting superior pharmacological effects and favorable bioavailability in CHF treatment [62]. Further research employed ultraperformance liquid chromatography coupled with quadrupole time‐of‐flight tandem mass spectrometry (UPLC‐Q‐TOF‐MS) to identify and validate key active constituents in QLQX, including ginsenosides Rg1 and Rb1, which constitute the major therapeutic components of QLQX against CHF [63]. Similarly, another study utilizing ultrahigh‐performance liquid chromatography coupled with triple quadrupole tandem mass spectrometry (UHPLC‐QqQ‐MS/MS) confirmed that six compounds, including ginsenoside Rb1, were the most prominent potential active ingredients in QLQX, providing critical insights for further research on its anti‐HF mechanisms [64].

### 4.3. Baoyuan Decoction (BYD)

BYD, a classical formulation consisting of ginseng (*P*. *ginseng* C.A. Mey.), milkvetch root (*A*. *membranaceus* [Fisch.] Bunge), cassia bark (*Cinnamomum cassia* [L.] J. Presl), and licorice (*Glycyrrhiza uralensis* Fisch. ex DC.), is clinically utilized in China for the treatment of aplastic anemia, chronic renal failure, and coronary heart disease. Pharmacokinetic studies employing liquid chromatography–mass spectrometry (LC‐MS) have identified several representative bioactive constituents in BYD, including ginsenoside Rb1, Re, Rd, Rg1 and flavonoids, which collectively contribute to its therapeutic effects against HF [65]. In a mechanistic study, BYD was evaluated using fosinopril (4.67 mg/kg) as a positive control in both in vitro and in vivo HF models. Cardiac ankyrin repeat protein (CARP), a novel biomarker and therapeutic target in HF, exacerbates cardiomyocyte apoptosis and cardiac dysfunction through angiotensin Type 1 (AT1) receptor activation. BYD was demonstrated to ameliorate HF and suppress myocardial apoptosis via modulation of the AT1‐CARP signaling pathway. Notably, key bioactive ginsenosides (Rg3, Rb1, Rc, and Re at 5 *μ*M) in BYD exhibited significant antiapoptotic effects [66]. In addition, recent research has further isolated and characterized four active compounds from BYD, including ginsenoside Rh2, to formulate a four‐compound mixture termed AGILe. Experimental evidence indicates that AGILe protects H9c2 cardiomyocytes from oxygen–glucose deprivation‐induced injury by targeting the TNF‐*α*/NF‐*κ*B pathway, suggesting its potential therapeutic utility in MI and cardioprotection [67].

### 4.4. Qixue‐Shuangbu Prescription (QSP)

QSP, a TCM formula designed to replenish both Qi and blood, typically comprises ginseng (*P*. *ginseng* C.A. Mey.), milkvetch root (*A*. *membranaceus* [Fisch.] Bunge), Chinese angelica (*Angelica sinensis* [Oliv.] Diels), and largehead atractylodes rhizome (*Atractylodes macrocephala* Koidz.). It has demonstrated efficacy in treating HF and myocardial ischemia. Pharmacokinetic studies in rats have identified ginsenosides Rg1 and Re as principal active components of QSP. Notably, the compound tincture formulation of QSP shows superior clinical efficacy compared with traditional decoction [68]. Using a doxorubicin‐induced CHF rat model, researchers employed microdialysis coupled with UPLC‐MS/MS to compare pharmacokinetic and pharmacodynamic profiles of processed versus crude QSP. The study validated the enhanced therapeutic effect of processed QSP in CHF treatment, particularly for seven bioactive components including ginsenosides Rb1, Re, and Rg1 [69].

### 4.5. Other Chinese Herbal Formulations

#### 4.5.1. Sheng Mai Yin (SMY)

SMY, a renowned herbal medicine in China composed of ginseng (*P*. *ginseng* C.A. Mey.), dwarf lilyturf tuber (*O*. *japonicus* [L.f.] Ker Gawl.), and Chinese magnolia vine fruit (*S*. *chinensis* [Turcz.] Baill.), is widely prescribed for cardiac conditions characterized by Qi and Yin deficiency syndrome. Ginsenoside Rg1 has been identified as a primary active constituent responsible for SMY′s anti‐HF effects. In a doxorubicin‐induced CHF rat model (2 mg/kg for 7 weeks), SMY (22.5–90 mg/kg)—standardized to contain ginsenoside Rg1 at 25.63 ± 3.42 − mg/g extract—demonstrated comparable efficacy to captopril (0.625 mg/kg) in ameliorating myocardial injury. Mechanistic studies revealed SMY attenuates CHF progression by suppressing pathological myocardial changes and cardiac remodeling, potentially through downregulation of IL‐6/TNF‐*α* and inhibition of MMPs/COL‐IV overexpression [70].

#### 4.5.2. Danqi Pill (DQP)

DQP, a TCM formulation composed of danshen root (*S*. *miltiorrhiza* Bunge) and sanqi root (*P*. *notoginseng* [Burkill] F.H. Chen), exhibits multicompound, multitarget characteristics in HF treatment. Through LAD‐induced HF mouse and OGD/R‐injured H9C2 cell models, ginsenoside Rb3 was identified as DQP′s primary active compound. Rb3 exerts cardioprotective effects by directly targeting RXR*α* to mitigate ROS‐induced metabolic dysfunction, maintaining mitochondrial homeostasis and improving energy metabolism [71].

#### 4.5.3. Qishen Yiqi (QSYQ) Dripping Pills

Clinically used for myocardial ischemic diseases, QSYQ, a formulation containing milkvetch root (*A*. *membranaceus* [Fisch.] Bunge), danshen root (*S*. *miltiorrhiza* Bunge), sanqi root (*P*. *notoginseng* [Burkill] F.H. Chen), and rosewood (*Dalbergia odorifera* T.C. Chen), exerts therapeutic effects through multitarget mechanisms, with the hypoxia‐inducible Factor 1 (HIF‐1) signaling pathway playing a pivotal role. Ginsenoside Rg1, a key active component, significantly downregulates HIF‐1*α* while upregulating the expression of vascular endothelial growth factor A, collectively protecting cardiomyocytes [72].

#### 4.5.4. Shenfu Injection (SFI)

Derived from the classical Shenfu decoction (SFD) consisting of red ginseng (*P*. *ginseng* C.A. Mey.) and prepared monkshood (*A*. *carmichaelii* Debeaux), SFI is clinically employed for HF and shock. UHPLC‐QqQ‐MS/MS quantification identified 18 ginsenosides (including Ra1‐3, Rb1‐3, Rc, Rd, Re, etc.) and aconite alkaloids as major active components in rat plasma, providing pharmacokinetic evidence for SFI′s clinical application [73].

#### 4.5.5. Er Shen Zhenwu Decoction (ESZD)

ESZD, a modified formulation based on Zhenwu decoction and typically containing ginseng (*P*. *ginseng* C.A. Mey.) and prepared monkshood (*A*. *carmichaelii* Debeaux) as key components, is a prescription for the treatment of CHF with yang deficiency of the heart and kidney. A study employing a rapid profiling strategy based on UHPLC‐QTOF‐MS has identified the major chemical constituents, including ginsenosides Rg1, Re, and Rb1. These compounds can serve as references for the quality control of ESZD and as potential therapeutic agents for the treatment of CHF [74].

#### 4.5.6. Xinbao Pill

With 30 years of clinical use for CHF, Xinbao pill, a formulation containing ginseng (*P*. *ginseng* C.A. Mey.), cassia bark (*C*. *cassia* [L.] J. Presl), prepared monkshood (*A*. *carmichaelii* Debeaux), and sanqi root (*P*. *notoginseng* [Burkill] F.H. Chen), improves cardiac function by reducing NT‐proBNP, mitigating myocardial injury, and degrading extracellular collagen. Three compounds including ginsenosides Rg1 and Rg2 constitute its active components, potentially acting through heme synthesis inhibition and succinyl‐CoA–mediated mitochondrial stabilization [27].

In conclusion, current evidence substantiates the therapeutic potential of ginsenoside‐containing Chinese herbal compounds in HF management. One or multiple ginsenosides serve as primary active components and pharmacodynamic markers in these formulations. For translational reference, the effective doses of these formulations in preclinical animal models typically range from 10 to 200 mg/kg/day, whereas the active ginsenoside constituents exhibit potency in vitro at concentrations commonly between 5 and 100 *μ*M. These findings provide both a theoretical foundation and clinical references for developing multitarget ginsenoside‐based combination therapies.

## 5. Clinical Research Progress

To date, clinical studies investigating the therapeutic effects of ginseng, ginsenosides, and their derivatives on HF remain relatively limited. Only a few clinical trials have reported the cardioprotective effects of ginseng in HF patients (Table 2), as summarized below.

**Table 2:** Clinical evidence of ginsenosides and ginseng‐containing TCM in heart failure management.

Study type	Intervention	Sample size (groups)	Endpoints/outcomes	Key findings	Limitations	Reference
RCT	Shenfu decoction	40 patients (intervention: 18; control: 22)	Quality of life and hepatic function	Adjuvant therapy significantly improved QoL and hepatic dysfunction in CHF patients.	Small sample size, short duration (14 days)	[75]
RCT	Shenmai injection	240 randomized (intervention: 120; placebo: 120)	Primary: NYHA class; secondary: 6MWD, SF‐36, LVEF, and BNP	Enhanced cardiac function when combined with standard therapy; recommended for CHF with CAD.	Short‐term (7 days), long‐term effects unknown	[76]
RCT	Xinyue capsule	426 total (intervention: 211; control: 215)	Congestive HF, stroke, and ACS readmission	Improved clinical outcomes (e.g., reduced HF readmission rates).	Highly specific population and no isolated CHF data	[77]
Systematic review	Shengmai injection	20 RCTs (*n* = 1562)	Cardiac function (LVEF, BNP) and safety	Improved cardiac function with good safety profile.	High risk of bias, Significant heterogeneity	[78]
Meta‐analysis	Ginseng‐containing TCM	28 studies (7 databases)	Clinical outcomes in acute decompensated HF (mortality and readmission)	Ginseng TCM combined with Western therapy showed superior outcomes.	Variable study quality and limited reliability	[79]

Abbreviations: 6MWD, 6‐min walking distance; ACS, acute coronary syndrome; BNP, B‐type natriuretic peptide; CAD, coronary artery disease; CHF, chronic heart failure; HF, heart failure; LVEF, left ventricular ejection fraction; NYHA, New York Heart Association functional classification; QoL, quality of life; RCT, randomized controlled trial; SF‐36, Short Form Health Survey; TCM, traditional Chinese medicine.

An early randomized controlled trial (RCT) evaluated the effects of SFD on quality of life and liver function in symptomatic CHF patients. The study enrolled 40 patients who were randomly assigned to either the SFD group (*n* = 18) or the control group (*n* = 22). Both groups received standard HF therapy, with the SFD group additionally receiving oral SFD for 14 days, whereas the control group received a placebo. The results demonstrated that adjuvant SFD treatment significantly improved both quality of life and liver injury in CHF patients compared with standard therapy alone [75]. Another RCT investigated the efficacy and safety of Shenmai injection (SMI) in CHF patients with comorbid coronary artery disease. The trial included 240 eligible patients, with New York Heart Association functional class as the primary endpoint, and secondary endpoints including 6‐min walking distance, Short‐Form 36 Health Survey score, left ventricular ejection fraction, and BNP levels. The findings suggested that integrative therapy combining standard medication with SMI further enhanced cardiac function in CHF patients, supporting the use of SMI as an adjunctive treatment for CHF with coronary artery disease [76]. In a study exploring herbal interventions for acute coronary syndrome (ACS) patients with renal dysfunction after percutaneous coronary intervention, Xinyue capsule, composed of ginsenosides extracted from ginseng leaves and stems, demonstrated significant improvements in clinical outcomes, including reduced risks of congestive HF, stroke, and ACS‐related rehospitalization [77]. Additionally, a systematic review of 20 RCTs evaluated another ginseng‐based formulation, Shengmai injection (SGMI), for CHF treatment. The authors concluded that SGMI, as an adjunct to conventional Western medicine, could improve cardiac function with a favorable safety profile. However, due to the high risk of bias in included trials and substantial heterogeneity in outcomes, these findings require validation through larger, high‐quality RCTs [78]. A recent systematic review and meta‐analysis assessed ginseng‐containing TCM for acute decompensated HF, analyzing 28 studies from seven databases. The results indicated that ginseng‐containing TCM provided significant benefits over conventional Western therapy alone. Nevertheless, the conclusion was limited by inconsistent trial quality, necessitating further rigorous studies for robust evidence [79].

Therefore, current clinical evidence on ginseng and ginsenosides for HF treatment remains limited by small sample sizes, few RCTs, and variable study quality. Future high‐quality research is essential to validate the cardioprotective effects of ginseng and its bioactive ginsenosides, thereby providing stronger evidence for their clinical translation and optimal therapeutic application in HF management.

## 6. Safety Profile and Potential Toxicity of Ginsenosides

Although the multitarget efficacy of ginsenosides in HF is promising, a critical appraisal of their safety profile is essential for clinical translation, especially given the complex medication regimens of HF patients. The general safety of orally administered ginseng is well‐documented, with adverse effects typically being mild, dose‐dependent, and reversible, including insomnia, headache, and gastrointestinal disturbances [80]. However, the primary safety concern for ginsenosides in the clinical management of HF is the potential for herb–drug interactions. Ginsenosides can modulate key drug‐metabolizing enzymes (e.g., cytochrome P450 3A4) and transporters (e.g., P‐glycoprotein), potentially altering the pharmacokinetics and effects of conventional HF drugs [81]. Notably, clinical and pharmacokinetic evidence suggests that ginseng can diminish the anticoagulant effect of warfarin. This interaction, initially flagged in systematic safety reviews [82], has been specifically confirmed in analyses of herbal medicine–warfarin interactions [83], highlighting a critical risk for thromboembolic events in comedicated patients. Although the limited clinical trials of ginseng‐based formulations in HF reported favorable safety profiles [76, 78], they were not designed to systematically detect such pharmacokinetic interactions.

Special attention is required for TCM formulations containing processed aconite (*A*. *carmichaelii* Debeaux), such as QLQX capsule and SFI. Aconite alkaloids, even after detoxification processing, possess potent cardiotoxic and neurotoxic properties primarily due to the persistent activation of voltage‐gated sodium channels by compounds such as aconitine [84]. The safe clinical use of these formulations therefore hinges on stringent quality control to limit these toxic alkaloids [85].

Currently, dedicated toxicological studies evaluating ginsenosides specifically for HF applications are lacking. Significant gaps exist in understanding their chronic toxicity, teratogenic potential, and comprehensive interaction profiles with cornerstone HF therapies like ARNIs, SGLT2 inhibitors, and beta‐blockers. Future preclinical development must prioritize these safety assessments. Concurrently, well‐designed clinical trials should incorporate robust pharmacokinetic interaction studies and systematic safety monitoring to establish a clear risk–benefit profile for ginsenoside‐based therapies in HF.

## 7. Conclusions and Perspectives

HF represents a complex clinical syndrome with multifaceted pathophysiology. Although early interventions for cardiac dysfunction can reduce HF risk, the limitations of single‐target therapies have spurred interest in multitarget strategies. However, as reviewed in the preceding section, the successful translation of these promising preclinical findings hinges not only on their multitarget efficacy but equally on a comprehensive understanding and rigorous assessment of their safety profile, including potential herb–drug interactions and the specific risks associated with complex formulations.

Ginseng, a traditional Chinese herb, has a long history of use in cardiovascular diseases [86, 87]. Ginsenosides, its primary active components, exhibit multitarget and multipathway protective effects against HF, providing a strong theoretical foundation for clinical applications. For instance, ginsenosides Rb1, Rb3, and Rg1 can suppress cardiomyocyte apoptosis and optimize myocardial energy metabolism by activating signaling pathways such as PI3K‐Akt and PPAR*α*, thereby alleviating HF [17, 20, 46]. Additionally, Rg3 has been shown to improve Ca^2+^ homeostasis and regulate myocardial pyruvate metabolism in HF treatment [29, 50]. Compound ginsenoside preparations have demonstrated synergistic effects in animal models, suggesting superior therapeutic advantages over single‐component treatments. Furthermore, the synergistic interactions between ginsenosides and conventional HF drugs (e.g., ARNIs, SGLT2 inhibitors), along with advancements in novel drug delivery systems such as nanoformulations, have expanded their clinical potential. However, current research still faces several translational challenges: most evidence is derived from cell or animal studies; clinical trials remain scarce, with small sample sizes and a lack of standardized protocols; the mechanisms of different ginsenoside monomers vary, and their pharmacokinetic properties and optimal combination ratios require further elucidation. Moreover, the combined efficacy and potential drug interactions between ginsenosides and conventional HF medications warrant further investigation.

**Figure 2:**
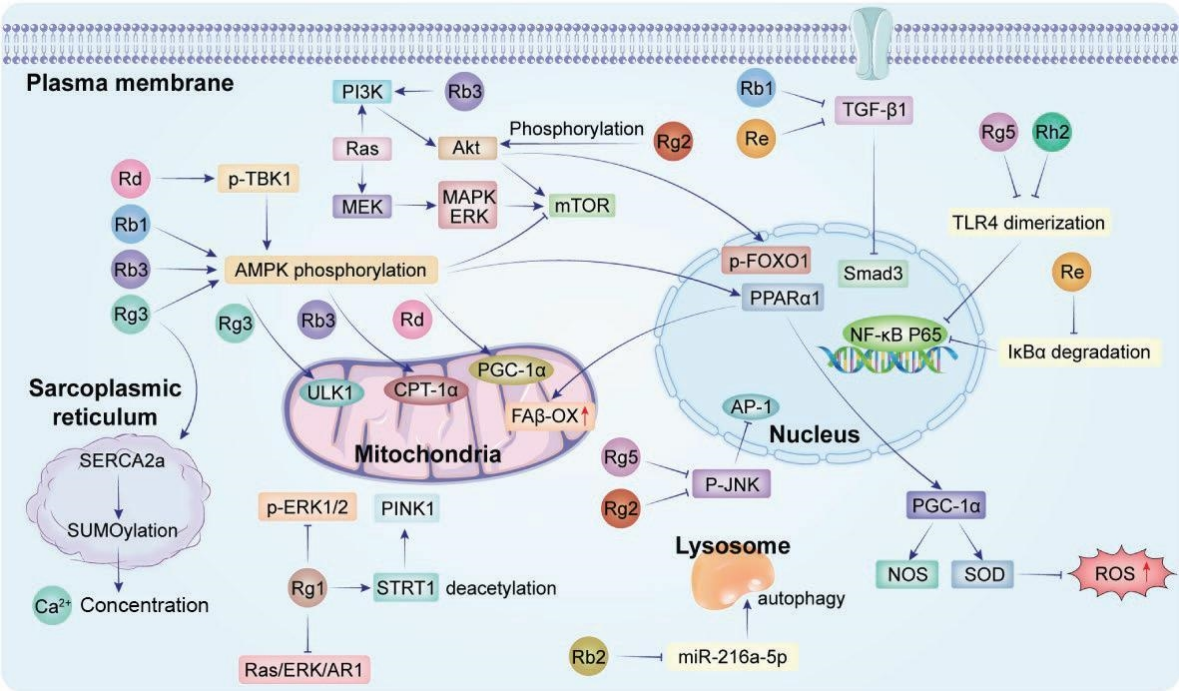
Key signaling networks underlying the antiheart failure effects of major ginsenosides.

This review systematically summarizes the multitarget protective mechanisms of ginseng and its active components, ginsenosides, in HF treatment, demonstrating their ability to improve cardiac function by regulating key pathways including metabolism, oxidative stress, fibrosis, inflammation, and apoptosis (Figure 2). These findings not only deepen the understanding of the “Fuzheng Guben” (supporting vital qi and strengthening body resistance) theory in TCM, but also provide scientific rationale for developing novel anti‐HF drugs. Although current research faces translational challenges requiring further validation through large‐scale clinical trials with standardized protocols, the pleiotropic characteristics of ginsenosides make them promising as complementary or alternative therapies for refractory HF, particularly suitable for patients with poor tolerance or inadequate response to existing medications. Future research should enhance the therapeutic value of ginsenosides by [1] deepening mechanistic studies using multiomics technologies and gene editing tools to elucidate their multitarget effects on cardiomyocyte homeostasis and complex signaling networks; [2] promoting clinical translation through large‐scale, multicenter randomized controlled trials to establish the efficacy, safety, and optimal dosing regimens of ginsenoside‐based preparations; [3] investigating synergistic effects between ginsenosides/their derivatives and conventional HF drugs (e.g., ARNIs and SGLT2 inhibitors) to develop integrated treatment strategies combining traditional and Western medicine; and [4] optimizing pharmaceutical innovations by improving bioavailability through structural modifications or nano‐carrier systems to overcome their low oral absorption and enhance therapeutic potency.

In conclusion, as naturally derived multitarget drug candidates, ginsenosides show broad prospects in HF treatment. With deeper integration of basic and clinical research, they are expected to transition from traditional applications to precision medicine, providing better options for HF patients worldwide. Through continuous innovation, ginsenoside‐based formulations may become important strategies for improving patient quality of life and advancing cardiovascular therapeutics.

**Nomenclature**
HF: heart failure
ARNI: angiotensin receptor‐neprilysin inhibitors
SGLT2i: sodium‐glucose cotransporter‐2 inhibitors
Ang II: angiotensin II
LAD: left anterior descending
eNOS: endothelial NO synthase
NO: nitric oxide
ISO: isoproterenol
CHF: chronic heart failure
NT‐proBNP: N‐terminal pro‐B‐type natriuretic peptide
TAC: transverse aortic constriction
MAPK: mitogen‐activated protein kinase
ACY1: aminoacylase‐1
ULK1: Unc51‐like Kinase 1
CFs: cardiac fibroblasts
OGD/R: oxygen–glucose deprivation/reoxygenation
YQFM: YiQiFuMai injection
TCM: traditional Chinese medicine
QLQX: Qiliqiangxin capsule
Q‐markers: quality markers
BYD: Baoyuan decoction
CARP: cardiac ankyrin repeat protein
AT1: angiotensin Type 1 receptor
QSP: Qixue‐Shuangbu prescription
SMY: Sheng Mai Yin
DQP: Danqi pill
QSYQ: Qishen Yiqi dripping pills
HIF‐1: hypoxia‐inducible Factor 1
SFD: Shenfu decoction
SFI: Shenfu injection
ESZD: Er Shen Zhenwu decoction
RCT: randomized controlled trial
NYHA: New York Heart Association functional classification
LVEF: left ventricular ejection fraction
6MWD: 6‐min walking distance
SF‐36: Short Form Health Survey
QoL: quality of life
SMI: Shenmai injection
ACS: acute coronary syndrome
SGMI: Shengmai injection

## Author Contributions

L.Z., J.S., and F.Y. conceived this study and wrote the manuscript. L.Z., Y.L., and W.Y. developed the search strategy. L.Z., W.Y., X.G., and J.S. provided methodological advice. F.Y. and X.G. revised the manuscript. All authors have reviewed this protocol.

## Funding

This study was supported by the Scientific Research Fund Project of Education Department of Yunnan Province (2024J0277), Yunnan Health Training Project of High Level Talents (H‐2025053), and Hengrui Research Fund of Kunming Medical University (YQHR2025‐Y10).

## Disclosure

All authors approved the final manuscript.

## Conflicts of Interest

The authors declare no conflicts of interest.

## Data Availability

No datasets were generated or analyzed during the current study.
